# Mandible elemental composition and mechanical properties from distinct castes of the leafcutter ant *Atta laevigata* (Attini; Formicidae)

**DOI:** 10.1098/rsfs.2023.0048

**Published:** 2024-04-12

**Authors:** Valentin Birkenfeld, Stanislav N. Gorb, Wencke Krings

**Affiliations:** ^1^ Department of Functional Morphology and Biomechanics, Zoological Institute, Christian-Albrechts-Universität zu Kiel, Am Botanischen Garten 1–9, 24118 Kiel, Germany; ^2^ Department of Cariology, Endodontology and Periodontology, Universität Leipzig, Liebigstraße 12, 04103 Leipzig, Germany; ^3^ Department of Electron Microscopy, Institute of Cell and Systems Biology of Animals, Universität Hamburg, Martin-Luther-King-Platz 3, 20146 Hamburg, Germany; ^4^ Department of Mammalogy and Palaeoanthropology, Leibniz Institute for the Analysis of Biodiversity Change, Martin-Luther-King-Platz 3, 20146 Hamburg, Germany

**Keywords:** Young's modulus, hardness, mouthparts, nanoindentation, biomechanics, cuticle

## Abstract

Leafcutter ant colonies are divided into castes with the individuals performing different tasks, based mostly on size. With the mandibles, the small minims care for the brood or the fungus, whereas the larger minors and mediae cut and transport plant material, with the ant size positively related to the material size. The mechanical properties and composition of the mandible cuticle have been previously tested in the soldiers as the largest caste, revealing that the cutting edges contained high contents of the cross-linking transition metal zinc (Zn). With regard to the smaller castes, no data are present. To study how the mandible size and function relates to its mechanical properties, we here tested the mandibles of minims, minors and mediae by nanoindentation. We found that the hardness (H) and Young's modulus (E) values increased with increasing ant size and that the mandible cutting edges in each caste have the highest H- and E-values. To gain insight into the origins of these properties, we characterized the elemental composition by energy-dispersive X-ray analysis, revealing that minors and mediae possessed higher content of Zn in the cutting edges in contrast to the minims containing significantly less Zn. This shows, that Zn content relates to higher mechanical property values. Additionally, it shows that all of these parameters can differ within a single species.

## 1. Background

The mandibles are important tools for Formicidae, as their versatility in applications, such as nest building, brood care, defence and hunting contributes to the success of the colony [1]. The success of ants and other social insects is also attributed to the division of labour among colony workers of different castes (e.g. [2]). This can be associated with morphological adaptations as muscle and mandible size and shape, leading to improved performance in their respective roles [2–10].

The emergence of castes can be influenced by the size of the colony [11] as for example observed in Attina ants. Most species that cultivate fungi as a food source for the colony are predominantly monomorphic and use substrates, such as faeces, decaying plant material, and insect carcasses for their fungi. By contrast, species with more individuals (*Atta* and *Acromyrmex*) exhibit a broader dietary spectrum, using fresh plant material, like leaves, branches and flowers for their fungi and display pronounced polyethism and polymorphism [11–13]. Their specialized symbiosis with fungus allows colonies to reach sizes of up to millions of individuals per colony, leading to the differentiation into various castes. *Atta* ants possess minims, minors, mediae and majors, with a broad and continuous size spectrum [14,15]. These different castes perform different tasks depending on their size [11,16,17]. The founding of an *Atta* colony is claustral, i.e. the queen does not need to leave her freshly dug and sealed nest (claustral chamber), since she can supply the fungus, brood, and herself with her bodily reserves including flight muscles and trophic eggs. While founding a new colony, *Atta* queens initially produce only workers capable of performing multiple tasks. These first workers have a head width of 0.8–1.6 mm and are mostly involved in brood care, even in mature colonies. Later with the colony development, workers with a head width of 0.8–1.0 mm are necessary for fungal gardening, while a head width of 1.6 mm on average is required for cutting tough leaves. Throughout colony growth, the spectrum extends in both directions, with small workers having a head width of less than 0.7 mm and large workers exceeding 5 mm in head width, increasing the range of used plant tissues [6]. The quantity of small workers in growing colonies is significantly higher than that of large workers and the size range is more dependent on colony size than age [14].

During cutting, the mandibles of the larger ants perform different actions. One mandible anchors itself in the plant material, while the other remains motionless. Then, the mandible with the plant item is moved towards the other one, which leads to the cutting of the material. Both mandibles can perform both tasks, depending on the cutting direction [18]. Once plant pieces are brought into the nest, smaller workers further shred the pieces, add droplets of faeces and shape them into spherical masses. Finally, even smaller workers plant the substrate mass with fungal hyphae, which are then tended by the smallest and most common workers. However, the executed tasks also depend on the age of the individuals, with young workers (callows) caring for nest duties and older ones for tasks outside the nest. This is related to the ontogeny of the bite apparatus (head capsule and muscles), as callows can generate less force with their mandibles than mature workers [7]. Task allocation, on the other hand, is also influenced by polymorphism, with the larger majors being more effective in defending against vertebrates and smaller workers in defending against foreign ants [14,19,20].

The mandibles are part of the insect exoskeleton and thus cuticle-based. The endo- and exocuticle are composite materials consisting of chitin fibres with associated proteins, whereas the epicuticle consists of proteins and lipids (see review by Vincent & Wegst [21]). The chitin fibrils are visible as lamellar layers, usually more tightly packed in the exocuticle than in the more hydrated endocuticle (e.g. [22]). The mechanical properties of the cuticle can vary significantly between different regions and these heterogeneities determine the functions of specific structures in addition to the morphology. Whether a region is hard and stiff or soft and flexible depends on the thickness of the cuticle, the orientation of the chitin fibres, the degree of chitin fibre linkage through sclerotization, or the distribution of proteins and water [23–27].

Another mechanism for hardening and stiffening the exocuticle is the incorporation of transition metals such as copper (Cu), iron (Fe), manganese (Mn) and zinc (Zn) ([23,28–34]; see reviews by Liu *et al*. [35] and Politi *et al.* [36]). These metals were identified as cross-links as they bind strongly to the biopolymers and increase the cross-linking density [35,37–41]. Additionally, there are indications for the involvement of biomineralization in the insect cuticle with alkaline earth metals such as calcium (Ca) and magnesium (Mg) [34,42–46]. In previous studies on ants (also in leafcutter ants), Zn was detected in the cuticle of the mandible cutting edge [5,47–50]. During ontogeny, Mn is first present in the cutting edges but is then replaced by Zn, the content of which increases more than 200-fold from a freshly emerged to a fully grown worker, making the region more than twice as hard [48]. In *Atta laevigata* (F. Smith, 1858) [51], the mandibles of soldiers were previously tested and Zn was identified in the cutting edges, presumably reducing wear [52]. Even though transition metals are present, the mandibles still become worn as documented for *Atta cephalotes*, which increases the time and energy needed to cut leaves and leading to a shift of performed tasks from leaf cutting to transportation as wear progresses [53].

Since *Atta* ants possess different castes performing different tasks, it is likely that the mandibles not only differ in size and shape, but also in material composition and mechanical parameters (hardness H and Young's modulus E). The present study aims to determine the material properties and composition of the mandibles of different *Atta laevigata* castes (minims, minors, mediae). First, mandible morphologies were documented by scanning electron microscopy (SEM). Then, the mechanical properties (hardness, Young's modulus) were tested by nanoindentation and the proportion of transition and alkaline earth metals was identified by energy-dispersive X-ray spectroscopy (EDX).

## 2. Material and methods

### 2.1. Animals

The specimens of *Atta laevigata* were originally obtained from a private animal breeding facility (online shop Myants.de, Weiden, Germany) in 2020. The colony used had roughly 8000 individuals. The ants were fed with fresh blackberry leaves, rose petals from Kiel and occasionally other leaves such as *Ligustrum*. Species identification was verified with the assistance of Prof. Jonathan Z. Shik, University of Copenhagen, Denmark. Only adult ants with fully hardened cuticles were used in this study.

**Figure 1.**
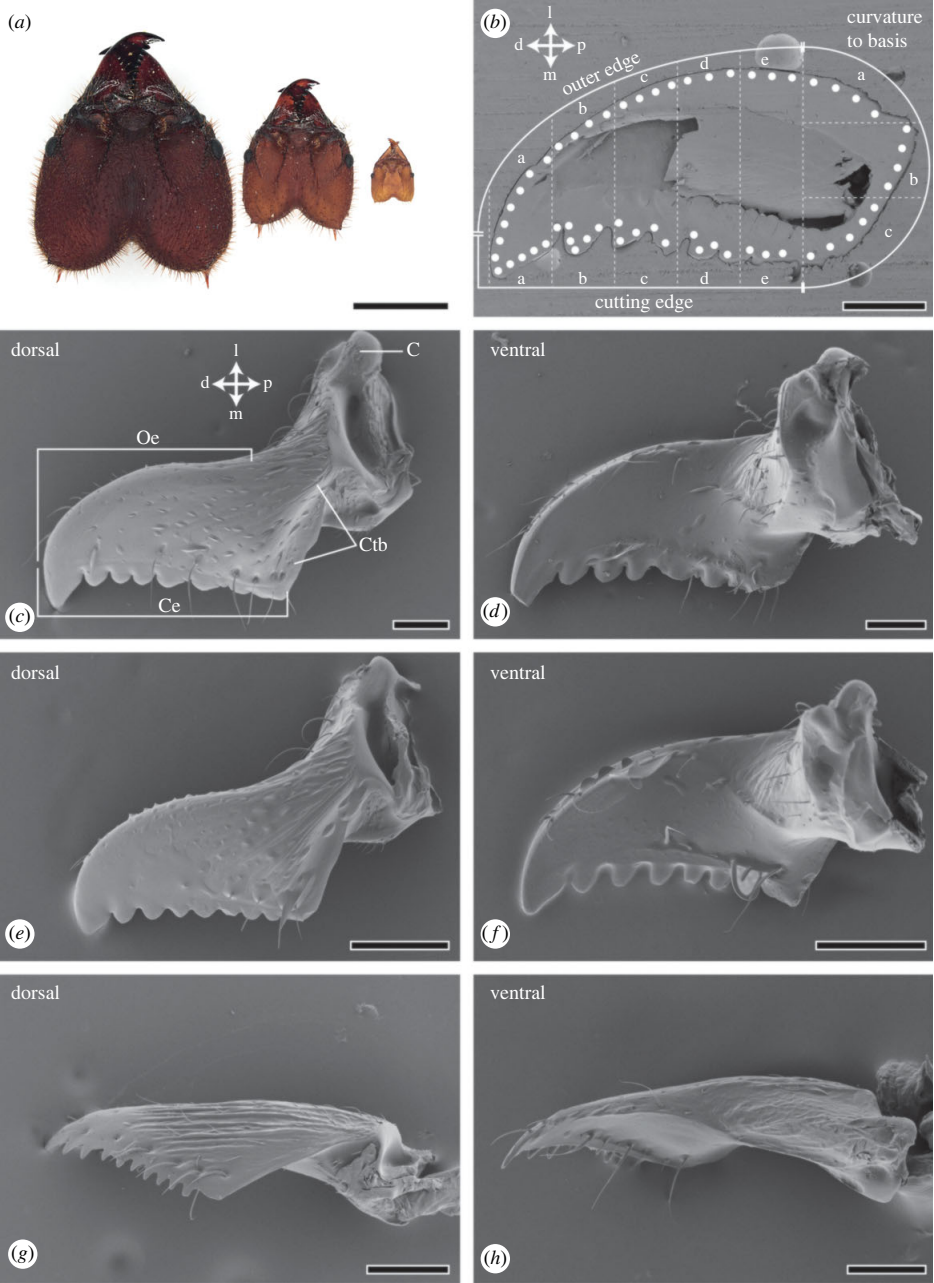
(*a*) Light microscopic images of one head from each caste (media, minor, minim). (*b*) Embedded and polished mediae mandible used for the EDX and nanoindentation experiments. The white spots highlight the localities of these tests at the exocuticle. The mandible was divided into three different regions (cutting edge, outer edge, curvature to the base). Each region was divided into three or five areas (a, b, c, d, e) and tested from distal (left) to proximal (right). (*c,d*) SEM images of one media mandible in dorsal and ventral view. (*e,f*) SEM images of one minor mandible in dorsal and ventral view. (*g,h*) SEM images of one minim mandible in dorsal and ventral view. Abbreviations: C, condyle; Ce, cutting edge; Ctb, curvature to basis; d, distal; p, proximal; m, medial; l, lateral; Oe, outer edge. Scale bars: *a*, 2 mm; *b–f*, 300 µm; *g–h* 100 µm.

For experiments, the individuals were categorized into the following castes, based on the literature [15]: ants with a head of 0.9–1.1 mm were classified as minims, those of 2.0–2.2 mm as minors and those of 3.7–3.9 mm as mediae (figure 1*a,c–h*). The studied colony was too small to possess soldiers (majors) as none could be identified in comparison with the literature [54].

Six ants from each size category were selected from the colony, killed using chloroform, and their mandibles dissected. The mandibles were then cleaned in 70% EtOH with an ultrasonic cleaner for 10 s. All mandibles were also documented employing a Keyence Digital Microscope VHX-7000 (KEYENCE, Neu-Isenburg, Germany) with automatic photo stacking software.

### 2.2. Scanning electron microscopy

For documentation of the morphology, the mandibles of one specimen per caste were glued to SEM sample holders using double-sided adhesive carbon pads (one in dorsal and one in ventral view). Subsequently, the samples were sputter-coated with a 5 nm thick layer of platinum. A Zeiss LEO 1525 (One Zeiss Drive, Thornwood, USA) was used for visualization; images were captured with 5 kV. Distances and magnifications were always adjusted for the best possible image quality.

### 2.3. Preparation for nanoindentation and elemental analyses

Mandibles of five specimens per caste were attached to glass microscope slides with double-sided adhesive carbon pads. A metallic ring was placed around each mandible and epoxy resin (Reckli Epoxy WST; RECKLI GmbH, Herne, Germany) was poured into each ring until the mandibles were completely covered. After 3 days of polymerization at room temperature, the samples were polished with sandpaper of different roughness to reach the cuticle areas of interest (cutting edge, outer edge, curvature to the base; figure 1*b*). Then samples were polished with 1 µm aluminium oxide powder (Presi, 00 120 180; PRESI GmbH, Hagen, Germany) on a polishing plate employing a polishing machine (Minitech 233/333; PRESI GmbH, Hagen, Germany). Afterwards, samples were cleaned in 70% EtOH for 5 min with an ultrasonic cleaner.

### 2.4. Energy-dispersive X-ray spectroscopy and nanoindentation

The embedded samples (*N* = 30 mandibles from 15 specimens; five specimens per caste) were used for energy-dispersive X-ray spectroscopy (EDX/EDS). For this purpose, the samples were attached to SEM sample holders with double-sided adhesive carbon pads and sputter-coated with a 5 nm thick layer of platinum. A Zeiss LEO 1525 equipped with an Octane Silicon Drift Detector (SDD) (Microanalysis system TEAM; EDAX Inc., New Jersey, USA) was used. All samples were measured with the same settings (acceleration voltage of 20 kV, working distance 15 mm, lens aperture 60 µm, measurement time for each measurement point 30 s, resolution 137.6 eV) following previous studies [55,56]. Calibration was performed with Cu (therefore, the results are semi-quantitative). Overall, 788 small areas (thereof 164 from minims, 264 from minors and 360 from mediae) on the mandible exocuticle sections (figure 1*b*) were tested (size 2 × 2–10 × 10 µm). The following elements were detected and analysed: aluminium (Al), carbon (C), calcium (Ca), sodium (Cl), copper (Cu), fluorine (F), iron (Fe), potassium (K), magnesium (Mg), manganese (Mn), nitrogen (N), sodium (Na), oxygen (O), phosphorus (P), platinum (Pt), sulfur (S), silicon (Si), zinc (Zn). We only took measurements with peaks of the respective elements that were higher than the noise, into account. Some elements were not discussed as they are either a part of chitin and the associated proteins (H, C, N, O) or could be artefacts from the polishing paste (Al, O). P and Pt (from the coating) were evaluated together because their overlapping peaks cannot be distinguished from one another. The platinum coating was however important to verify the analysis (i.e. if P + Pt was found in very high proportions, e.g. greater than 5 atomic %, this one measurement was excluded from results). For some analyses, Ca, Cl, Cu, F, Fe, K, Mg, Mn, Na, P, Pt, S, Si, and Zn were summed up as ‘all elements (Ae)’.

For nanoindentation, the samples (*N* = 30 mandibles from 15 specimens; five specimens per caste) were glued to sample holders following established protocols [55,56]. Each sample was tested at room temperature along the mandible exocuticle, with an average of 25–50 indentations per sample, using an SA2 Nanoindenter (MTS Nano Instruments, Oak Ridge, TN, USA) equipped with a Berkovich diamond tip. In total, 871 mandible localities (thereof 123 from minims, 271 from minors and 477 from mediae) were tested.

### 2.5. Statistics

All statistical analyses were conducted using JMP Pro, Version 14 (SAS Institute Inc., Cary, USA). Means and standard deviations were calculated. To determine normality, a Shapiro–Wilk *W*-test was performed. Since the data were not normally distributed, a Kruskal–Wallis test was conducted. Groups were compared pairwise with Wilcoxon method. Furthermore, correlations between parameters were calculated in JMP Pro and, in part, visualized with Excel, version 16.0 (Microsoft Corporation, Redmond, Washington, USA).

## 3. Results

### 3.1. Size

There were clear size differences between the mandibles from the three castes (figure 1*c–h*). The mandibles of the mediae (figure 1*c,d*) were larger, wider, possessing larger teeth, and a greater degree of curvature, compared to those of the minors (figure 1*e,f*). The same was true for the minors in comparison to the minims (figure 1*g,h*). The length of the cutting edge was approximately 1.3 mm for the mediae, about 730 µm for the minors, and approximately 210 µm for the minims.

### 3.2. Material composition

**Figure 2.**
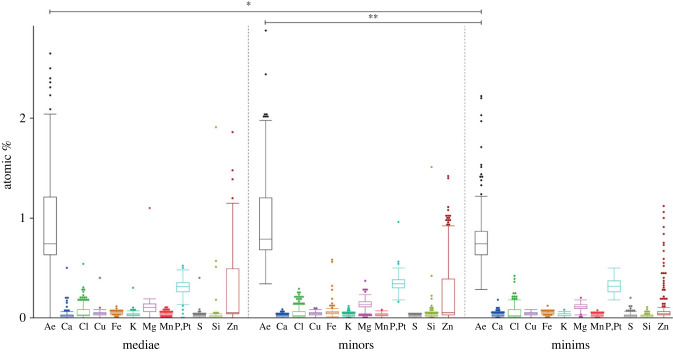
Results of EDX analyses: proportions of the individual elements and Ae (sum of Ca, Cl, Cu, Fe, K, Mg, Mn, P + Pt, S, Si, Zn), given in atomic %, sorted to the castes.

The mandibles of mediae possessed highest content of Ae (sum of Ca, Cl, Cu, Fe, K, Mg, Mn, P + Pt, S, Si, Zn), followed by the ones of minors and minims (figure 2 and, for values, see electronic supplementary material, table S1). Mandibles of mediae and minims as well as of minims and minors were significantly different with regard to Ae (for *p*-values, see electronic supplementary material, table S2).

The following elements were distributed in the mandibles in the following order, sorted to their descending mean values (in atomic %) (figure 2 and, for values, see electronic supplementary material, table S1): mediae: P + Pt, Zn, Mg, Cl, Fe, Cu/K, Mn/Ca/Si and S; minors: P + Pt, Zn, Mg, Fe, Cu/Cl, K/Mn/Si, Ca and S; minims: P + Pt, Zn/Mg, Cl, Cu/Fe, Ca/K/Mn and Si/S.

With regard to Ca, Fe, K, Mg, Mn, P + Pt, S and Si, highly significant or significant differences were detected between mandibles of mediae and minors (for *p*-values see electronic supplementary material, table S2). Ca, Cl, K, Mn, S and Zn content differed significantly or highly significantly between mediae and minims; Ca, Cl, Fe, K, Mg, Mn, P + Pt, Si and Zn content differed highly significantly or significantly between mandibles of minors and minims (for *p*-values, see electronic supplementary material, table S2).

**Figure 3.**
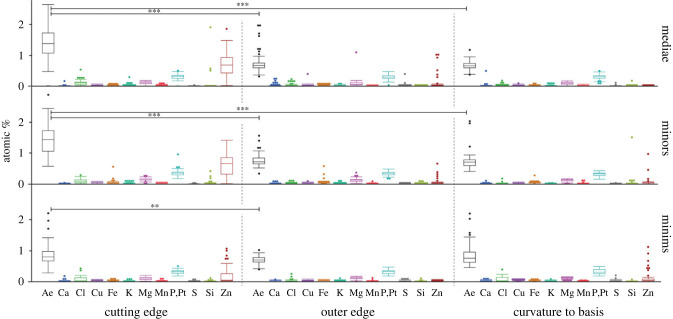
Results of EDX analyses: proportions of the individual elements and Ae (sum of Ca, Cl, Cu, Fe, K, Mg, Mn, P + Pt, S, Si, Zn), given in atomic %, sorted to the castes and regions (cutting edge, outer edge, curvature to the basis).

With regard to different mandible regions, we found that specifically Zn was abundant in the cutting edge in all castes, whereas the outer edges and the curvatures to the bases possessed very low Zn content (figure 3 and electronic supplementary material, table S1). In mediae and minors, the Zn content of the cutting edges were highly significantly different from the content of the outer edges and the curvatures (for *p*-values see electronic supplementary material, table S3). In minims, differences with regard to Zn were only detected between the cutting edges and the outer edges.

With regard to Ae, Ca, Mg, Mn and S, the cutting edges of mediae and minors were highly significantly different to the outer edges and the curvatures, whereas the curvatures and the outer edges were not different (for *p*-values, see electronic supplementary material, table S3). In minims, the cutting edges were highly significantly different to the outer edges and curvatures with regard to Ca and S.

Since the cutting edges were highly significantly different to the outer edges and the curvatures with regard to most elements, we here focused on them when comparing between castes. The cutting edges of minims and minors showed highly significant or significant differences with regard to Ae, Ca, Cl, Fe, Mg, Mn, P + Pt, Si and Zn (for *p*-values see electronic supplementary material, table S4). Cutting edges of minims and mediae were significantly or highly significantly different with regard to Ae, Ca, Cl, Mg, Mn, P + Pt and Zn. Minors and mediae were significantly different with regard to Cl, Fe, K, Mg and P + Pt, but not with regard to Ae, Ca, Cu, Mn, S, Si and Zn. Cutting edges of minims and mediae were not different with regard to Cu, Fe, K, S and Si and those of minims and minors with regard to Cu, K and S (for *p*-values see electronic supplementary material, table S4).

### 3.3. Mechanical properties

**Figure 4.**
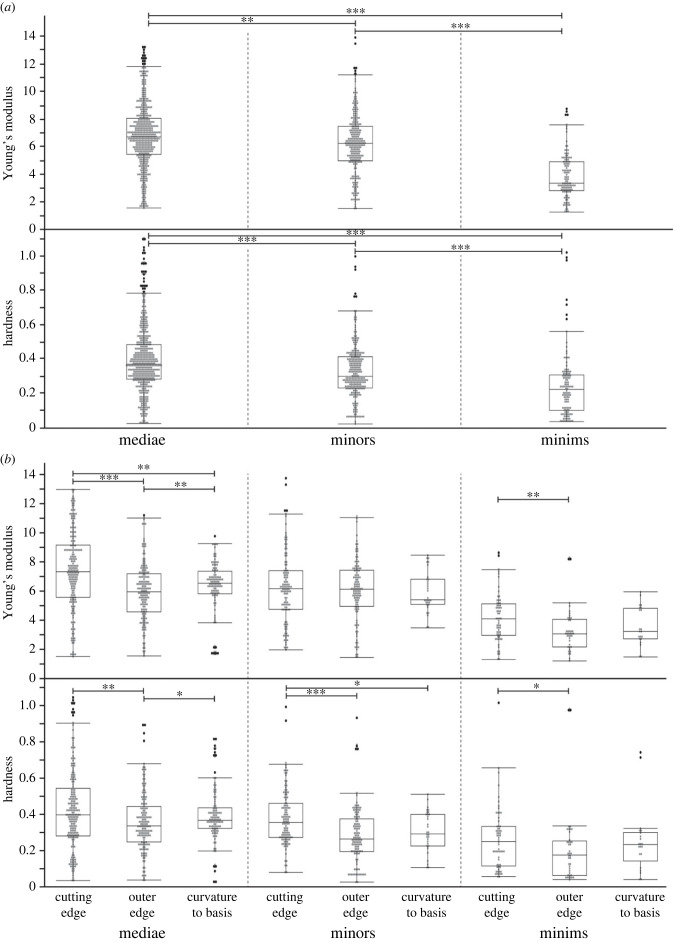
Results of nanoindentation: hardness and Young's modulus (both in GPa) for each caste. (*a*) Results from all regions and areas pooled together. (*b*) Results sorted to the regions (cutting edge, outer edge, curvature to the basis).

The values of hardness (H) ranged from approximately 0.02 to approximately 1.11 GPa and of stiffness (E, Young's modulus) from approximately 1.18 to approximately 13.70 GPa (figure 4). The mandibles from the mediae were highly significantly harder and stiffer than the mandibles from the minors (figure 4*a* and electronic supplementary material, table S5 for values; for *p*-values, see electronic supplementary material, table S2). The latter were highly significantly harder and stiffer than the mandibles of the minims (figure 4*a* and electronic supplementary material, table S5 for values; for *p*-values, see electronic supplementary material, table S2).

In each caste, the cutting edges were the hardest and stiffest localities, followed by the curvature and finally by the outer edge as the softest and most flexible region (figure 4*b* and electronic supplementary material, table S5 for values). In mediae, H values were highly significantly or significantly different between the curvature and the outer edge as well as the cutting edge and the outer edge, whereas the curvature and the cutting edge were not different (for *p*-values, see electronic supplementary material, table S3). In minors, the curvature and the cutting edge as well as the outer edge and the cutting edge were highly significantly or significantly different, whereas the curvature and the outer edge were not different. In minims, the outer edge and the cutting edge were significantly different. In mediae, E-values showed highly significant differences between all regions, whereas the E-values of the minors were not different (for *p*-values, see electronic supplementary material, table S3). In minims, the cutting edges were highly significantly different from the outer edges, whereas the cutting edge and the curvature as well as the outer edge and the curvature were not different. When regions were compared between the castes, we found that the cutting edges, the curvatures, and the outer edges were significantly or highly significantly different with regard to H and E (for *p*-values see electronic supplementary material, tables S6 and S7).

### 3.4. Correlations and relationships between parameters

All correlation coefficients can be found in the electronic supplementary material, table S8. We detected a high correlation between H and E (*r* = 0.85), Mn and Zn (0.79), Cl and Zn (0.72). A moderate correlation was determined for H and Mn (0.60).

When the ranges of the parameters were included in the analysis (see electronic supplementary material, figures S1–S16), H had a clear relationship with the content of Cl, Cu, Fe, Mn, Si and Zn (see electronic supplementary material, figures S2–S4, S6–S8, respectively). E was related to the content of Cu, Fe, Mn, Si and Zn (electronic supplementary material, figures S11, S12, S14–S16, respectively).

## 4. Discussion

### 4.1. Cuticle properties

The cuticle is a composite material consisting of chitin nanofibres embedded in a matrix of associated proteins [21]. The mechanical properties range from KPa to GPa and depend on the composition of the respective region and the water content ([57–59] see reviews by Vincent & Wegst [21], and Stamm *et al*. [60]). Structures like mouthparts, joints or claws, which are prone to abrasion, are harder (e.g. [23,33,48,61–65]) than structures that experience minimal wear (e.g. [58,59,66–71]).

With regard to mandibles, E values ranging from 4 to 20 GPa and H from 0.2 to 2.0 GPa were found in termites, dragonfly, antlion and beetle larvae [23,33,34,65]. In previous studies on *Atta cepholotes* and *A. sexdens*, the mandible cutting edges of larger ants were found to have maximum E-values of 5 GPa and maximum H values of 0.2 GPa [50], 0.5 GPa (VHN: 52 kgmm^−2^; see [47]), or 1.0 GPa [48]. For *Atta laevigata* soldiers, Brito *et al*. [52] determined the following values: for the cutting edge (internal region) E of 6 GPa and H of 0.36 GPa; for the outer edge (external region) E of 3 GPa and H of 0.19 GPa. In the present study, the values of E ranged from 1.18 to 13.70 GPa, and of H from 0.02 to 1.11 GPa, falling within the typical range of insect mandibles. The smallest mandibles (from minims) were the softest and most flexible ones, whereas the large mandibles (from mediae) were the hardest and stiffest ones. Following this, the mandibles of majors (soldiers) should be the hardest. We did not test majors, because none was present due to the smallness of the colony, but the published maximum values for soldier mandible cutting edges were E = 7.58 GPa and H = 0.58 GPa [52]. The highest values documented in this study for the mediae (large ants) were 13.70 GPa for E and 1.11 GPa for H, exceeding published results. However, due to variations in measurement conditions and sample preparation, direct comparisons with published results are difficult [60]. However, it could also be possible that soldiers possess thicker exocuticles, potentially compensating the smaller E and H values, but this, however, awaits further investigation.

Similar to previous studies [48,50,52], the cutting edges were here found to be the hardest part of the mandible, presumably because they experience the most interaction with materials, aiming to minimize wear and maintain cutting ability [53]. The curvature towards the base was harder than the outer edge in most individuals of this study, potentially because the curvature experiences higher stresses during cutting, transferring the stresses from the cutting edges towards the head capsule.

The mechanical property and size differences between the mandibles of the different castes can potentially explain the division of labour. Smaller workers (minims) perform tasks that do not require significant strength or exposure to high forces, e.g. the delicate handling of brood or caring for brood and fungus [14,19]. As worker size increases, so does the mandible size, width, curvature and tooth size. The teeth of the mediae and minors may be advantageous for anchoring in leaf surfaces and biting into potential threats. Minors usually cut softer plant material and transport it, whereas the mediae handle harder and thicker plant parts, which would be explained by the lower E and H values of minors. The opening angle of the large mandibles was found to generate higher bite forces [72], which means that mandibles experience higher stresses. Higher stress levels in the mandibles and head capsules of large *Pheidole* ants were previously identified by finite-element analyses, taking morphology into account [8–10]. For *Atta*, this was suggested based on the analyses of muscular and structural parameters [2] and was verified by bite force experiments [6,7]. The high E-values of the large workers (mediae) are likely an adaptation to reduce structural failure, when experiencing higher stresses, whereas the high H values probably reduce wear, when cutting challenging plant materials.

### 4.2. Origin of mechanical properties

The mechanical properties of the cuticle (see review by Politi *et al*. [36]) can result from the microstructure, the abundance of proteins as well as the degree of sclerotization by quinone reactions ([28,48,73–75]; for review on mechanical property gradients and their various origins, see [35]).

Even though the insect cuticle lacks a higher content of minerals, transition metals (Cu, Fe, Mn, Zn), with colocalized halogens (Cl) and alkaline earth metals (Ca, Mg) were additionally found to be abundant in structures prone to structural failure or wear, such as mouthparts [23,28,29,31–34,41,48,49,61,76–83]. Cu, Fe, Mn and/or Zn ions probably serve as cross-links [41,61,80,84–87], whereas Ca and Mg could be present in crystalline form [34,43–45]. Most of the elements, especially Zn, Mn, Ca and Mg, directly relate to an increase in hardness and wear resistance in insects [23,28,29,33,34,61,63,74,88,89].

In ants, transition metals such as Zn were previously also detected in the mandible cutting edges [5,49], also in *Atta* relating to higher hard and stiffness values [47,48,50,52].

In the present study, we detected Zn in the cutting edges, which were the hardest and stiffest regions in all castes. This supports the hypothesis that Zn in the cuticle increases E and H values presumably leading to a higher wear resistance and the reduction of structural failure. In mediae and minors, Zn was found to be abundant in higher proportions than Ca, Cu, Fe, Mg and Mn, whereas in minims, Zn content was low. Zn and Cl can be colocalized, as observed in the worm *Nereis* [86,87]. This also seems to be the case in the mandibles of *Atta laevigata*. However, it is worth noting that Mn also correlated with Cl and Zn.

## Data Availability

All results can be found in the electronic supplementary material [90].
